# Enhancing Interfacial Lithiophilicity and Stability with PVDF/In(NO_3_)_3_ Composite Separators for Durable Lithium Metal Anodes

**DOI:** 10.3390/nano14141229

**Published:** 2024-07-20

**Authors:** Zhuzhu Du, Xin Chen, Hongfang Du, Ying Zhao, Yuhang Liu, Wei Ai

**Affiliations:** 1 School of Materials Science and Engineering, Institute of Flexible Electronics and Intelligent Textile, Xi’an Polytechnic University, Xi’an 710048, China; 2 Frontiers Science Center for Flexible Electronics, Shaanxi Institute of Flexible Electronics, Northwestern Polytechnical University, Xi’an 710072, China; 3 Strait Laboratory of Flexible Electronics, Strait Institute of Flexible Electronics (Future Technologies), Fujian Normal University, Fuzhou 350117, China; 4 Xi’an Hongxing Electronic Paste Technology Co., Ltd., Xi’an 710199, China

**Keywords:** separator modification, polyvinylidene fluoride, Li-In alloy, Li_3_N, Li metal anodes

## Abstract

Separator modification is a promising method for advancing lithium metal anodes; however, achieving homogeneous lithium-ion flux and uniform plating/stripping processes remains challenging. In this work, we introduce a novel approach by developing a composite separator, termed PVDF-INO, which integrates In(NO_3_)_3_ (INO) into polyvinylidene fluoride (PVDF) to create a 12 μm thick layer. This addition significantly enhances the interaction between the separator and the electrolyte, creating a lithophilic matrix that ensures an even distribution of lithium ions. This uniform ion distribution promotes consistent lithium deposition and dissolution, resulting in a durable, dendrite-free lithium metal anode. Moreover, the PVDF-INO separator not only enhances the affinity with electrolytes but also maintains stable lithium-ion flux, which is essential for reliable and safe battery operation. Consequently, it sustains operation over 750 h in a Li||Li symmetric battery configuration, with a low overpotential of just 28 mV. Additionally, full cells equipped with LiFePO_4_ cathodes and the PVDF-INO separator exhibit superior cycling performance, maintaining a capacity retention of 92.9% after 800 cycles at 1 C. This work paves the way for significant advancements in the field of lithium metal batteries, offering a promising solution to longstanding energy storage challenges.

## 1. Introduction

Lithium-ion batteries are at the forefront of eco-friendly energy storage technologies, playing a pivotal role in reshaping the portable electronics and automotive sectors globally [1,2]. Despite their success, current batteries utilizing graphite anodes (theoretical capacity: 372 mAh g^−1^) fall short in satisfying the stringent requirements of grid-scale applications and electric vehicles [3,4]. This has spurred the exploration of next-generation batteries with higher energy densities. A promising approach is the use of lithium metal anodes (LMAs), which offer a superior capacity of 3860 mAh g^−1^ and an ideal electrochemical potential of −3.04 V versus the standard hydrogen electrode [5,6,7]. However, challenges such as excessive electrolyte consumption and the cracking or remodeling of the solid electrolyte interphase (SEI) layer arise from uncontrolled dendritic growth during lithium deposition and dissolution [8,9,10]. Addressing these issues necessitates the development of dendrite-free lithium plating processes. Strategies to mitigate these challenges have included anode modification, engineering of artificial SEI layers, construction of host with lithiophilic sites (e.g., functional groups, heteroatom dopants, metal or its compounds, etc.), optimized flow collection structures, electrolyte refinement, and separator functionalization [11,12,13,14]. By comparison, separators play a critical role in managing electrolyte movement, facilitating ion transport, and serving as electrical insulators between the cathode and anode, significantly influencing overall battery performance.

Polypropylene (PP), known for its chemical and electrochemical stability, has been the standard in commercial applications for decades [15,16]. Yet, its random pore structure and poor wettability with carbonate electrolytes often lead to dendritic growth, thereby shortening battery life [17,18]. To overcome these limitations, researchers have focused on tailoring the pore structure and enhancing the surface properties of separators [19,20]. Innovations such as PP coated with carbon materials, covalent organic frameworks, metal–organic frameworks, and lithium-containing compounds have shown potential in directing the flow of lithium ions, improving lithium plating behavior, and increasing lithiophilicity [21,22,23,24]. However, these methods often require additional space within the battery, involve labor-intensive and time-consuming manufacturing processes, and are not yet scalable for commercial production.

In this work, we introduce a novel composite separator, termed PVDF-INO, which consists of polyvinylidene fluoride (PVDF) integrated with In(NO_3_)_3_ (INO). The PVDF’s polar functional groups enhance the electrolyte’s wettability with LMA, ensuring a more uniform lithium-ion flux and reducing erratic lithium deposition. When the PVDF-INO separator contacts lithium metal, it forms an interfacial layer composed of Li-In alloy and Li_3_N through in situ displacement and redox reactions, significantly enhancing the interfacial lithiophilicity and stability. This organic/inorganic composite layer enables the stable cycling of LMA under high current densities and large capacities. Moreover, the full cells exhibit a robust cycling lifespan, enduring 800 cycles at 1 C (1 C = 170 mA g^−1^) while maintaining 92.9% capacity retention.

## 2. Results and Discussion

**Figure 1:**
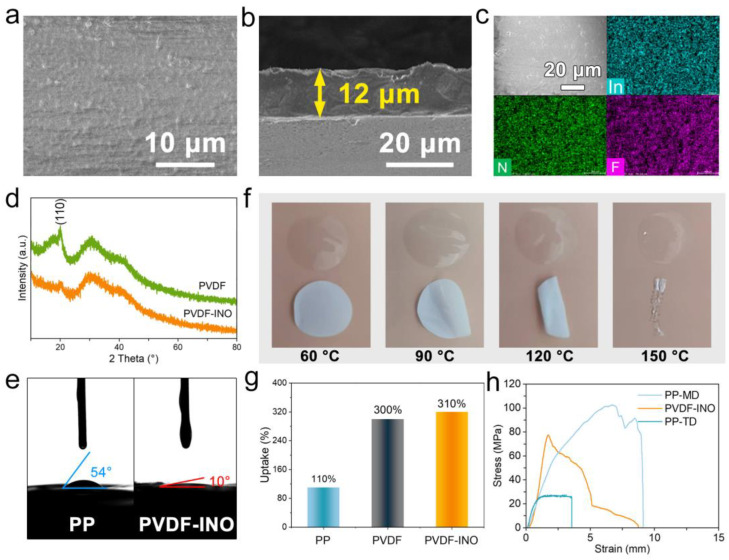
(**a**) Top-view and (**b**) cross-section SEM images of the PVDF-INO separator. (**c**) EDS mapping images of the PVDF-INO separator. (**d**) XRD patterns, (**e**) contact angle, (**f**) heat resistance, (**g**) electrolyte uptake rate, and (**h**) stress–strain curves of PP and PVDF-INO.

Figure S1a presents a scanning electron microscopy (SEM) image of a commercial PP separator, illustrating an irregular pore distribution. This irregularity typically leads to chaotic lithium-ion flow, resulting in uneven and rough electrodeposit layers on LMAs, ultimately hastening battery failure due to persistent lithium salt depletion [25,26]. In contrast, the PVDF-INO composite separator, produced through a scraping and coating method (Figure S2), features a smooth surface that facilitates the orderly transmission of lithium ions along the PVDF molecular chain, as depicted in Figure 1a. The inclusion of INO additives is based on the knowledge that NO_3_^−^ can induce the formation of a Li_3_N layer, while In^3+^ facilitates a rapid reaction to form the Li-In alloy interphase with high ion conductivity. The composite separator has a uniform thickness of approximately 12 μm (Figure 1b), thinner than that of commercial PP separator (20 μm, Figure S1b), which will significantly reduce the interfacial resistance and minimize the Li-ion transport pathways. Energy-dispersive X-ray spectroscopy (EDS) of the composite separator, shown in Figure 1c, indicates a uniform and dense distribution of the In, N, and F elements. This uniformity aids in forming a closely adherent SEI layer on the lithium electrode [27]. Fourier-transform infrared spectroscopy (FTIR) tests in Figure S3 corroborate this, with absorption peaks at 1650 cm^−1^ and 1280 cm^−1^ confirming the successful integration of the In(NO_3_)_3_ additive. X-ray diffraction (XRD) patterns in Figure 1d reveal characteristic PVDF peaks at 19° and 31°, corresponding to an α-phase. The introduction of In(NO_3_)_3_ slightly reduces peak intensity, indicating that the inorganic additive disrupts the crystallization process of the polymer, leading to more amorphous regions [28]. Wettability tests depicted in Figure 1e compare the contact angles of commercial PP and PVDF-INO composite separators. The commercial PP separator displays a contact angle of 54°, while the pure PVDF membrane shows a dramatically lower angle of 10°. This demonstrates the greater wettability of PVDF-INO composite membrane, attributed to the strong polarity of the C-F bonds that enhance electrolyte interaction [29,30]. Accordingly, the electrolyte transfer rate and ion conductivity in the PVDF-INO separator will be greatly improved, indicative of the improvement in the interfacial reaction kinetics and uniformity of Li-ion flux at the electrode surface. Thermal stability testing, as shown in Figure 1f, assesses the separators’ performance at elevated temperatures. Both the PVDF-INO and the PP separators retain their circular shape initially (60 °C). Upon gradual heating from 60 to 120 °C, the PP separator exhibits significant curling due to anisotropic tensions arising during manufacturing, and melts at 150 °C. The PVDF-INO composite separator effectively improves the heat resistance of the separator and avoids battery safety accidents caused by shrinkage of the separator when battery thermal runaway occurs. Figure 1g highlights the superior electrolyte absorption capability of the PVDF-INO separator over its PP counterpart, with a 310% electrolyte uptake rate. This is probably attributed to the more amorphous regions in the PVDF-INO separator, which are beneficial for electrolyte accessibility. Mechanical properties were evaluated through tensile strength tests (Figure 1h), with the results displayed in stress–strain curves. Owing to the effect of pore structure, the tensile performance of the PP separator changes orientation, where the tensile strength in the machine direction (MD) is higher than that in the perpendicular transverse direction (TD). In contrast, the PVDF-INO exhibits typical plastic deformation with a tensile strength of 78.7 MPa, in contrast to PP’s variable tensile strength across different directions, further highlighting its role in reducing Li dendrite formation variability.

**Figure 2:**
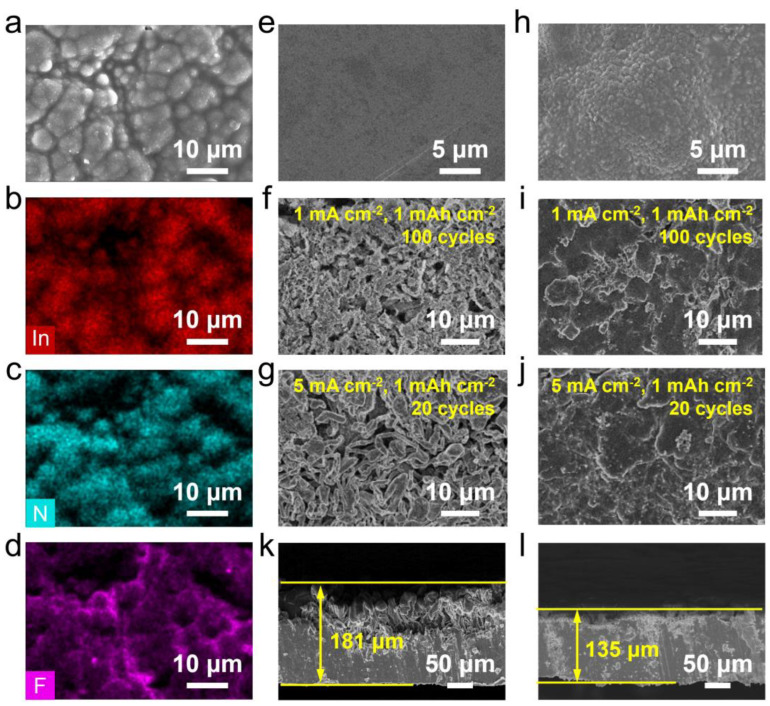
(**a**) SEM, and the corresponding (**b**) In, (**c**) N, and (**d**) F elemental mapping images of the lithium electrode from the batteries assembled using PVDF-INO separator. SEM images of the lithium electrodes (**e**) after reaction with the PP separator, and after cycling (**f**) for 100 cycles at 1 mA cm^−2^ and (**g**) for 20 cycles at 5 mA cm^−2^. SEM images of the lithium electrodes (**h**) after reaction with the PVDF-INO separator, and after cycling (**i**) for 100 cycles at 1 mA cm^−2^ and (**j**) for 20 cycles at 5 mA cm^−2^. Cross-section SEM images of the cycled lithium electrode with (**k**) PP and (**l**) PVDF-INO separators.

To evaluate the efficacy of the PVDF-INO composite separator in modifying the SEI layer of LMA, symmetrical batteries were disassembled after a 1 h stand period. EDS mapping reveals that the lithium metal surface is densely populated with uniformly distributed small particles, corresponding to an even dispersion of In, N, and F elements (Figure 2a–d). The uniform construction of In and N on the lithium surface results from the reaction between In(NO_3_)_3_ and metallic lithium upon contact, forming a Li-In alloy and Li_3_N. The Li-In alloy serves as a lithiophilic site to promote lithium deposition, while the high ionic conductivity of Li_3_N enhances the diffusion of lithium ions and mitigates side reactions on the electrode surface [31,32]. Further SEM images of the recycled electrodes provide concrete evidence that the PVDF-INO separator effectively mitigates the accumulation of inactive lithium. Figure 2e–g shows that after 100 cycles at 1 mA cm^−2^ and 20 cycles at 5 mA cm^−2^, respectively, loosely deposited structures with numerous lithium dendrites are present on the lithium anode surfaces covered by the PP separator. These dendrites can exacerbate electrolyte consumption, leading to safety risks such as internal shorts and thermal runaway in the battery. In stark contrast, the lithium anode covered with the PVDF-INO separator exhibits a relatively flat and dense surface devoid of visible Li dendrite formation (Figure 2h–j), indicating that the modified SEI layer possesses robust mechanical strength capable of withstanding rapid lithium plating/stripping cycles at high current densities without structural failure. To further investigate the PVDF-INO separator’s impact on suppressing lithium dendrite growth, cross-sectional SEM analysis of the lithium electrodes post-deposition was conducted. Figure 2k,l reveals that after 10 cycles at a current density of 1 mA cm^−2^ and a capacity of 5 mAh cm^−2^, the thicknesses of the lithium electrodes using the PP and PVDF-INO separators measure 181 and 135 μm, respectively. This comparison underscores that lithium metal paired with the PVDF-INO separator undergoes fewer volumetric changes during cycling, enhancing electrode integrity and potentially extending the battery’s lifespan.

**Figure 3:**
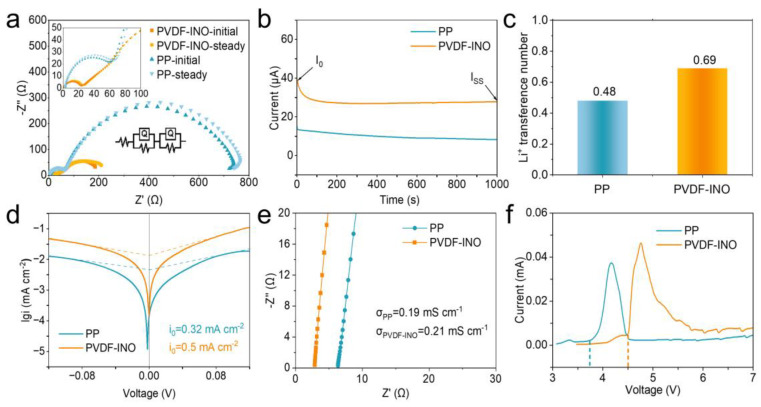
(**a**) EIS curves of the cells with PP and PVDF-INO separators at the initial and steady states. (**b**) I-t curves recorded with a polarization voltage of 10 mV. (**c**) Comparison of the t_Li+_ of PP and PVDF-INO. (**d**) Tafel profiles of the symmetric cells with PP and PVDF-INO separators. (**e**) EIS of the cells for determining the ionic conductivity. (**f**) LSV curves of the asymmetric cells.

Ion transport behaviors in PP and PVDF-INO separators were investigated through electrochemical measurements using the classical Bruce-Vincent method to determine the lithium-ion transference number (t_Li+_). During the experiment, both separators were incorporated into Li||Li symmetric batteries. The potentiostatic polarization process progressed from the initial state to a steady state, with interface resistances derived via electrochemical impedance spectroscopy (EIS) tests (Figure 3a, the inset shows the equivalent circuit model). Notably, the battery equipped with the PVDF-INO separator exhibits a lower SEI resistance compared to its PP counterpart. Upon applying a polarization voltage of 10 mV until the current-time curve stabilized, the t_Li+_ values for PP and PVDF-INO are calculated to be 0.48 and 0.69, respectively (Figure 3b,c). Furthermore, Tafel plots reveal that the exchange current densities for PVDF-INO and PP separators are 0.5 and 0.32 mA cm^−2^, respectively (Figure 3d). These findings indicate that the PVDF-INO separator facilitates faster lithium-ion migration and enhances electrochemical kinetics [27,33]. Ionic conductivity was measured for both separators in a cell configuration of stainless steel/separator/stainless steel using EIS (Figure 3e). The ionic conductivity of the PVDF-INO separator was found to be 0.21 mS cm^−1^, slightly higher than that of PP (0.19 mS cm^−1^), likely due to its improved wettability as discussed previously in Figure 1e. The electrochemical stability of the separators, soaked in electrolyte, was assessed via linear sweep voltammetry (LSV). As illustrated in Figure 3f, the potential window of the PVDF-INO separator reached 4.5 V (relative to Li^+^/Li), surpassing the 3.7 V of the PP separator. This suggests that the PVDF-INO separator offers better compatibility with carbonate-based electrolytes and may be more suitable for use in high-voltage and high-power batteries.

**Figure 4:**
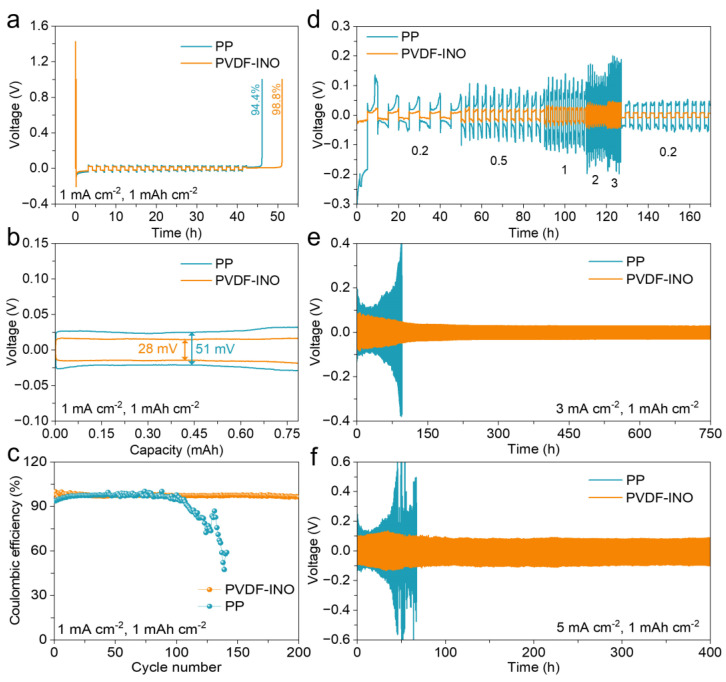
(**a**) Aurbach coulombic efficiencies of Li||Cu half-cell containing PP and PVDF-INO separators. (**b**) Capacity–voltage curve of Li||Cu half-cell assembled using PP and PVDF-INO separators. (**c**) Coulombic efficiencies of Li||Cu half-cells during 200 cycles. Cycling performance of the symmetric cells at (**d**) varying current densities, (**e**) 3 mA cm^−2^, and (**f**) 5 mA cm^−2^ with a capacity of 1 mAh cm^−2^.

Under testing conditions of 1 mA cm^−2^ and 1 mAh cm^−2^, the Aurbach coulombic efficiency of the battery equipped with the PVDF-INO separator consistently maintains 98.8% after 20 cycles, whereas the battery using the PP separator demonstrates only 94.4% (Figure 4a). The higher efficiency with the PVDF-INO separator can be attributed to the reduced consumption of both electrolyte and lithium metal within the cell. Figure 4b illustrates the capacity-voltage curve, highlighting lithium metal deposition/dissolution behavior. The Li||Cu half-cell containing the PVDF-INO separator exhibits a lower polarization voltage of 28 mV compared to 51 mV for the cell with the PP separator. After 200 cycles, the battery with the PVDF-INO separator maintains an average coulombic efficiency above 98%, in contrast to the cell with the PP separator, which shows a notable decline to below 90% by the 100th cycle (Figure 4c). These findings indicate that the PVDF-INO separator effectively suppresses lithium dendrite formation and enhances the cycling stability of the lithium anode. EIS plots of batteries with different separators before and after 50 cycles were analyzed to examine changes in interface resistance (Figure S4). The SEI resistance of the Li||Li battery with the PP separator before and after cycling is determined to be 74 and 48 Ω, respectively. In contrast, the Li||Li battery using the PVDF-INO separator shows much lower SEI resistances of 25 and 18 Ω, demonstrating the beneficial effects of the Li_3_N-enriched SEI layer on accelerating electrochemical reactions.

Further tests on Li||Li symmetric cells under varying current densities were conducted to explore the impact of the PVDF-INO separator on the lifespan of the LMAs and its efficiency in facilitating ion transport (Figure 4d). By increasing the current density from 0.2 to 3 mA cm^−2^, the voltage polarization in the cell using the PVDF-INO separator increases only by 60 mV, compared with 180 mV for the PP separator, indicating faster electrode kinetics with the PVDF-INO separator. At a current density of 3 mA cm^−2^, the symmetric cells with the PVDF-INO separator could be cycled stably for 750 h with a minimal hysteresis voltage of 28 mV (Figure 4e). Conversely, the cells with the PP separator exhibit a rapid increase in hysteresis voltage after just 50 h of cycling, leading to battery failure within 100 h due to an excessive growth of lithium dendrites and an accumulation of inactive lithium [34,35]. At an increased current density of 5 mA cm^−2^, the rapid lithium plating/stripping cycles severely damage the lithium electrode in the cells with the PP separator, rendering them unsuitable for high-rate cycling (Figure 4f). In contrast, cells with the PVDF-INO separator demonstrate stable operation even after 400 cycles, maintaining a hysteresis voltage of about 70 mV. These results underscore the superior interface properties of the PVDF-INO separator and its effectiveness in inhibiting dendrite growth.

**Figure 5:**
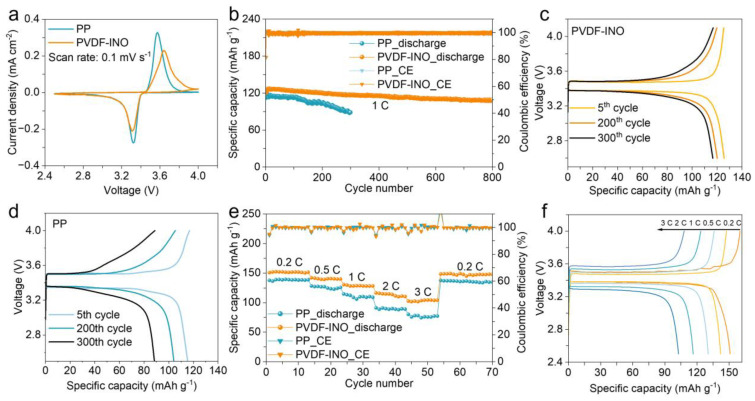
(**a**) CV curves of the LFP||PVDF-INO||Li and LFP||PP||Li cells at a scan rate of 0.1 mV s^−1^. (**b**) Cycling stability, and the corresponding (**c**,**d**) voltage–capacity curves of the LFP||PVDF-INO||Li and LFP||PP||Li cells at 1 C. (**e**) Rate performance and (**f**) voltage–capacity curves of the LFP||PVDF-INO||Li and LFP||PP||Li cells under varying current rates.

Full cells were assembled using LiFePO_4_ (LFP) as the cathode material, which is a typical olivine crystalline phase [36,37,38]. Figure 5a displays the cyclic voltammetry (CV) curves for both LFP||PVDF-INO||Li and LFP||PP||Li cells, where the integral area under the curve for the PP separator during the positive sweep is larger than that for the PVDF-INO, suggesting greater electrolyte decomposition in the PP-based cells. This demonstrates that the PVDF-INO separator significantly enhances the oxidation resistance of the electrolyte. While the slight peak shift may be attributed to the Li insertion/extraction in Li-In alloy, at a discharge rate of 1 C, the battery incorporating the PVDF-INO separator exhibits a specific capacity of 109 mAh g^−1^ after 800 cycles, achieving a capacity retention of 92.9% (Figure 5b). In contrast, the battery using the PP separator only maintains a capacity of 88 mAh g^−1^ after 300 cycles, with a capacity retention of 75.2%. Figure 5c,d presents the polarization voltage curves of the batteries with different separators, highlighting that the LFP||PVDF-INO||Li configuration not only sustains a higher capacity but also exhibits a lower polarization voltage. Furthermore, the specific capacities of the LFP||PVDF-INO||Li battery at various rates (0.2, 0.5, 1, 2, and 3 C) consistently exceed those of the LFP||PP||Li battery, with the disparity in discharge capacity becoming more pronounced as the rate increases (Figure 5e,f). In addition, the PVDF-INO separator is also coupled with the high-voltage LiCoO_2_ (LCO) cathode to evaluate the compatibility, and the assembled LCO|| PVDF-INO||Li full cell delivers a stable cycling for 100 cycles at a current density of 100 mA g^−1^, maintaining a high-capacity retention of 92.5% (Figure S5a). The corresponding highly overlapped voltage curves, as depicted in Figure S5b, indicate a steady polarization voltage and a reversible Li plating/stripping during long-term cycling. The superior performance of the PVDF-INO separator in lithium metal batteries is attributed primarily to its rapid ion transport capabilities and the uniform distribution of lithium-ion flux.

## 3. Conclusions

In summary, a composite PVDF-INO separator comprising PVDF and In(NO_3_)_3_ was developed. The separator’s abundant polar functional groups in PVDF facilitate rapid lithium-ion transport, while its uniform thickness and compositional distribution promote consistent and balanced lithium-ion flux. This design significantly reduces the interfacial resistance between electrode and electrolyte, and it enables a higher lithium-ion transference number of 0.69, an ionic conductivity of 0.21 mS cm^−1^, and a wide potential window of 4.5 V. Additionally, the In(NO_3_)_3_ additive within the separator reacts in situ with the LMA to form an SEI layer rich in Li-In alloy and Li_3_N, thereby inducing reversible and uniform lithium metal deposition and stripping. This layer adheres closely to the lithium metal surface, effectively preventing direct contact between the electrolyte and lithium metal, thereby mitigating interface reactions with the electrolyte. The Li||Li battery equipped with the PVDF-INO composite separator demonstrates an exceptional lifespan, operating up to 750 h without short-circuiting. Moreover, the LFP||PVDF-INO||Li battery exhibits significantly enhanced cyclic performance, maintaining a capacity retention rate of 92.9% after 800 cycles at 1 C. This work introduces an innovative separator design strategy that effectively distributes lithium-ion flux, offering substantial potential in extending the lifespan of lithium metal batteries.

## Data Availability

The data that support the findings of this study are available from the corresponding author upon reasonable request.

## Supplementary Materials

The following supporting information can be downloaded at: https://www.mdpi.com/article/10.3390/nano14141229/s1, Figure S1. (a) Top-view and (b) Cross-section SEM images of the PP separator. Figure S2. (a) Schematic diagram for the preparation of PVDF-lNO separator. (b) Photograph of PVDF-INO separator coated on a glass plate. Figure S3. FT-IR spectra of the PP, PVDF and PVDF-INO. Figure S4. EIS plots of the cells assembled with (a) PP and (b) PVDF-INO separators, both before and after cycling. Figure S5. (a) Cycling stability and the corresponding (b) voltage-capacity curves of the LCO ||PVDF-INO||Li full cells at a current density of 100 mA g^−1^.
