# Supplementary material for: Enhancing Interfacial Lithiophilicity and Stability with PVDF/In(NO3)3 Composite Separators for Durable Lithium Metal Anodes
Source: Nanomaterials (Basel). 2024 Jul 20;14(14):1229. doi: 10.3390/nano14141229 (PMC11279910; doi:10.3390/nano14141229)
Supplement: Supplementary file 1 [file nanomaterials-14-01229-s001.zip › nanomaterials-3089382-supplementary.pdf]

Supporting Information

Enhancing Interfacial Lithiophilicity and Stability with PVDF/In(NO_3)$_3$ Composite Separators for Durable Lithium Metal Anodes

Zhuzhu Du, [a,b] Xin Chen, [b] Hongfang Du, [c] Ying Zhao, [d] Yuhang Liu [b]* and Wei Ai [b]*

[a] School of Materials Science and Engineering, Institute of Flexible Electronics and Intelligent Textile, Xi'an Polytechnic University, Xi'an 710048, China
[b] Frontiers Science Center for Flexible Electronics, Shaanxi Institute of Flexible Electronics, Northwestern Polytechnical University, Xi'an 710072, China
[c] Strait Laboratory of Flexible Electronics, Strait Institute of Flexible Electronics (Future Technologies), Fujian Normal University, Fuzhou 350117, China
[d] Xi'an Hongxing Electronic Paste Technology Co., Ltd., Xi'an 710199, China
* Correspondence: liuyh@mail.nwpu.edu.cn, iamwai@nwpu.edu.cn

1. Experimental Section

1.1. Fabrication of PVDF-INO separator

5 g of PVDF was dissolved in 5 mL of N,N-dimethylformamide under stirring at 60 °C. Subsequently, 500 mg of In(NO_3)$_3$ was added to the solution. After all the solids dissolved, the slurry was spread onto a glass plate and heated at 60 °C to evaporate the solvent. The resultant film was then peeled off and cut into circular shapes with a diameter of 18 mm.

1.2. Material characterizations

SEM images were collected using a scanning electron microscopy (Verios G4) at an accelerating voltage of 3 kV. XRD patterns were measured on a Bruker D8 advance diffractometer with Cu Kα radiation. Contact angles were obtained usinga drop shape analyzer system (DSA1005).

1.3. Electrochemical measurements

All cells were assembled in an argon-filled glovebox with water and oxygen levels below 0.1 ppm, and subsequently tested using a NEWARE battery testing system. The electrolyte is 1 M $LiPF_6$ in EC:DEC (1:1, v/v) containing 5% FEC, with each cell receiving 60 μL. PVDF-INO and celgard 2500 (PP, Guangdong Canrd New Energy Technology Co.,Ltd) materials were employed as separators. The PP separator has a thickness of 20 μm, the porosity of 55% and an average pore size of 0.064 μm.

For the Li||Cu half cells were configured with Cu and lithium serving as the cathode and anode, respectively. Coulombic efficiency (CE) tests were firstly conducted by the Aurbach's methods. Concretely, a fixed capacity of 2 mAh cm^{-2} (Q_{T1}) was firstly plated on the Cu electrode, and then the cells were cycled for n cycles at the 1 mAh cm^{-2} (Q_C) for 1 mA cm^{-2} until the cut-off voltage increased to 1 V (Q_{S1}), which can calculate the Aurbach CE according to the following Equation (1):

$$CE = \frac{nQ_C + Q_{S1}}{nQ_C + Q_{T1}} \tag{S1}$$

The conventional CE tests were then performed based on half cells, in which the cells were firstly precycled at 0.2 mA cm^{-2} for 5 cycles in 0-1 V before cycling. Then, CE tests were performed by plating a fixed amount of Li (Q_{T2}) and then stripped until the cutoff

voltage increased to 1 V (Q_{S2}). Therefore, the average CE can be calculated according to the following Equation (2):

$$CE = \frac{1}{n}\sum\frac{Q_{T2}}{Q_{S2}} \quad \text{(S2)}$$

For the Li||Li cells, lithium foil was used for both the anode and cathode. In the assembly of full cells, $LiFePO_4$ (LFP, an average particle size of 1.13 μm, a specific surface area of 9.91 m^3 g^{-1} and purity of 99.9%, Guangdong Canrd New Energy Technology Co.,Ltd) or $LiCoO_2$ (LCO, an average particle size of 6.75 μm, a specific surface area of 0.14 m^3 g^{-1} and purity of 99.9%, Guangdong Canrd New Energy Technology Co.,Ltd) with an areal loading of 3 mg cm^{-2} served as the cathode. The slurry was obtained by blending LFP or LCO, acetylene black, and polyvinylidene fluoride (8:1:1 in weight) in N-methylpyrrolidone, which was then spread onto a carbon-coated aluminum foil and dried overnight at 60 °C under vacuum. The Li-LFP and Li-LCO full cells were tested within a potential range of 2.5-4.0 V and 2.5-4.3 V, respectively.

2. Supplementary Figures

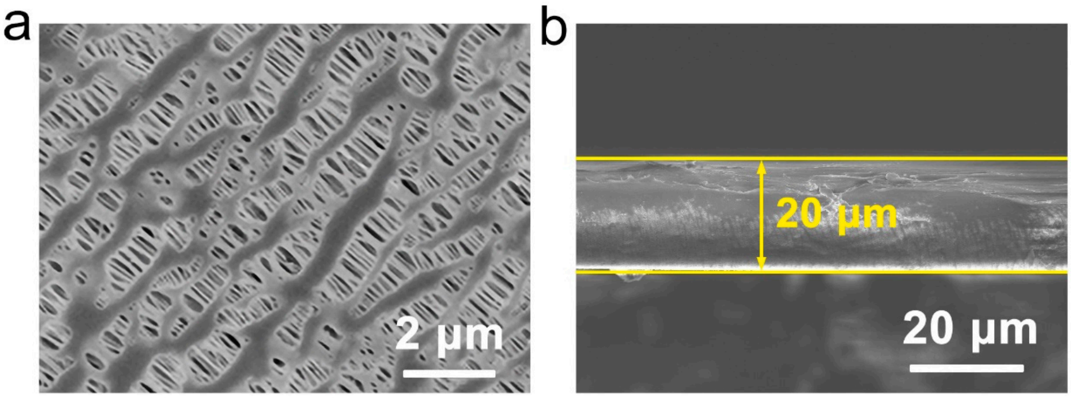

Figure S1. (a) Top-view and (b) Cross-section SEM images of the PP separator.

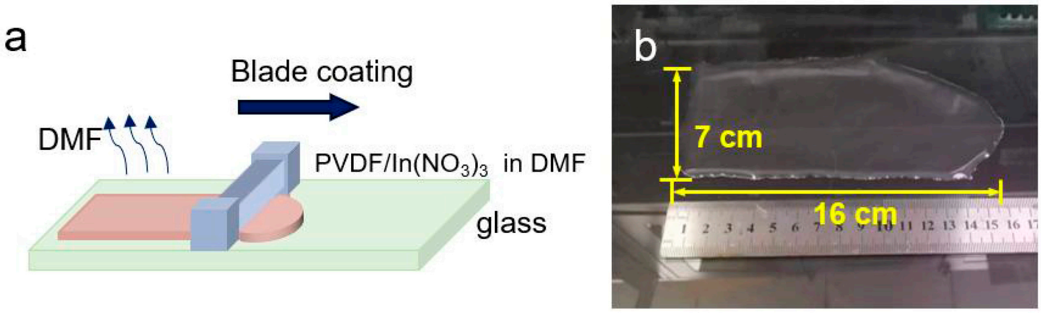

Figure S2. (a) Schematic diagram for the preparation of PVDF-INO separator. (b) Photograph of PVDF-INO separator coated on a glass plate.

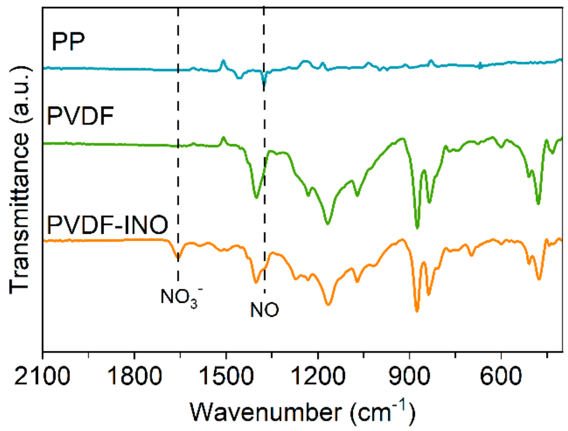

Figure S3. FT-IR spectra of the PP, PVDF and PVDF-INO.

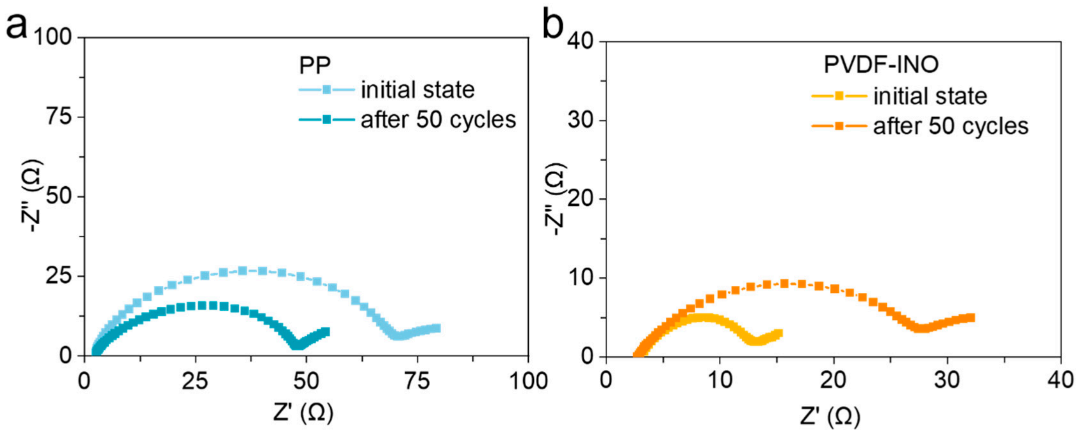

Figure S4. EIS plots of the cells assembled with (a) PP and (b) PVDF-INO separators, both before and after cycling.

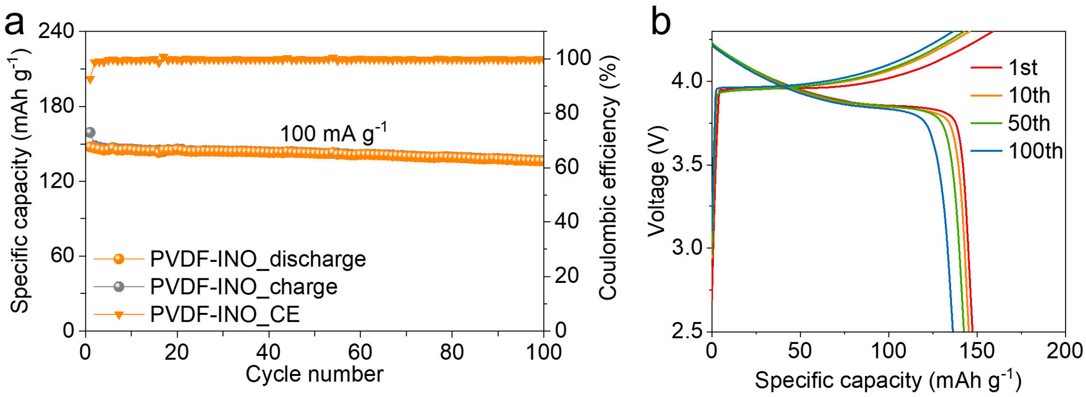

Figure S5. (a) Cycling stability and the corresponding (b) voltage-capacity curves of the LCO ||PVDF-INO||Li full cells at a current density of 100 mA g^{-1}.
